# Emergence of Resonances in Neural Systems: The Interplay between Adaptive Threshold and Short-Term Synaptic Plasticity

**DOI:** 10.1371/journal.pone.0017255

**Published:** 2011-03-08

**Authors:** Jorge F. Mejias, Joaquin J. Torres

**Affiliations:** 1 Center for Neural Dynamics, University of Ottawa, Ottawa, Ontario, Canada; 2 Department of Electromagnetism and Physics of the Matter, University of Granada, Granada, Spain; Mount Sinai School of Medicine, United States of America

## Abstract

In this work we study the detection of weak stimuli by spiking (integrate-and-fire) neurons in the presence of certain level of noisy background neural activity. Our study has focused in the realistic assumption that the synapses in the network present activity-dependent processes, such as short-term synaptic depression and facilitation. Employing mean-field techniques as well as numerical simulations, we found that there are *two* possible noise levels which optimize signal transmission. This new finding is in contrast with the classical theory of stochastic resonance which is able to predict only one optimal level of noise. We found that the complex interplay between adaptive neuron threshold and activity-dependent synaptic mechanisms is responsible for this new phenomenology. Our main results are confirmed by employing a more realistic FitzHugh-Nagumo neuron model, which displays threshold variability, as well as by considering more realistic stochastic synaptic models and realistic signals such as poissonian spike trains.

## Introduction

It is known that a certain level of noise can enhance the detection of weak input signals for some nonlinear systems. This phenomenon, known as stochastic resonance (SR), is characterized by the presence of a peak, or a bell-shaped dependence, in some information transfer measurement as a function of the noise intensity [1]–[3]. More precisely, for low noise levels the system is not able to detect the signal due to its small amplitude. For moderate noise levels, however, the noise is able to enhance the signal up to a certain detection threshold, and this makes the system respond in a highly correlated fashion with the signal (and a peak of information transfer appears). Finally, for too high noise levels the output is dominated by the noise and the signal is not detected.

Stochastic resonance has been measured in a wide variety of physical and biological systems, including bidirectional ring lasers [4], electronic circuits [5], crayfish mechanoreceptor [6], or voltage-dependent ion channels [7]. In the brain, it has been found in different types of sensory neurons [8], [9], in the hippocampus [10], in the brain stem [11], and in some cortical areas [12]–[15]. Although SR behavior has been extensively studied in many works, most of them assume a controlled source of noise that affects the dynamics of the system additively and, in some cases, without temporal correlations. Such assumption is no longer valid in *in vivo* experiments in actual neural systems, where noise is the result of the inherent activity of the medium (which could be, for instance, the highly irregular spontaneous activity of other cortical regions projecting to the structure of interest) and, therefore, not easily controlled by the experimentalist. The effect of such stochasticity on the dynamics of a particular neuron could, indeed, involve details concerning concrete biological mechanisms not considered yet. In particular, since neurons receive signals from its neighbors though synapses, the concrete characteristics of synapses may strongly influence the SR properties of *in vivo* neural circuits. It is known, for instance, that actual synapses present activity dependent mechanisms, such as short-term depression (STD) and short-term facilitation (STF), that may strongly modify the postsynaptic neural response in a nontrivial way. The former of these mechanisms considers that the amount of neurotransmitter ready to be released – due to the arrival of an action potential (AP) – is limited, and the synapse needs some time to recover these resources in order to transmit the next incoming AP. Synaptic facilitation, on the other hand, has an opposite effect and increases the postsynaptic response under repetitive presynaptic stimulation. Such increment is mediated by the influx of calcium ions into the presynaptic terminal [16]. The competition between STD and STF may be highly relevant in signal detection in noisy environments, as for instance in cortical gain control [17] or in spike coincidence detection [18], and therefore they could have a main role in SR tasks.

In addition to these synaptic mechanisms, the dynamics of the neuron firing threshold constitutes another important issue to be considered in the study of SR phenomena in neural media. In particular, it has been recently found that the firing threshold of cortical neurons may be raised as a consequence of a slow depolarization due to an input current [19], [20]. The biophysical origin of such effect is believed to be the differential kinetics of the voltage-dependent activation and inactivation of sodium and potassium channels on the neuron membrane (for further details, see [19]). Fast depolarizations due to input fluctuations, on the other hand, do not cause such effect [20], [21]. This increment of the firing threshold with slow depolarizations, which has been found in different cortical regions in vivo, is known as adaptive threshold dynamics. A prominent feature of such dynamics is that, unlike other neural adaptation mechanisms (see [22], [23]), adaptive threshold dynamics allows a neuron to raise its firing threshold without the generation of previous APs. In cat striate cortex, for instance, the existence of adaptive neural thresholds seems to play an important role in stimulus orientation by reducing cellular sensitivity to slow depolarizations [19], [21]. It is highly relevant, therefore, to take into account the possible effect of such mechanism in signal detection in noisy environments. Indeed, high cortical activity levels could cause slow depolarizations and affect the excitability properties of the neuron, and therefore its information transmission properties, via adaptive threshold dynamics. The mechanism of adaptive threshold dynamics has been captured by a number of neuron models [24]–[28]. However, the complex interplay between dynamic synapses and adaptive thresholds has caught little attention from researchers, despite the computational implications that it may have in SR properties of actual neural systems.

In this work, we use a phenomenological model of dynamic synapses and a standard integrate-and-fire (IF) neuron model with an input-dependent threshold to study the interaction between adaptive threshold, STD and STF in the detection of weak (subthreshold) signals under a noisy environment. More precisely, we consider a system of 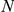 presynaptic neurons which transmit APs, within a Poisson distribution with mean frequency 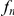, to a postsynaptic neuron through dynamic synapses. In these conditions, a weak and low-frequency sinusoidal signal is also transmitted to the postsynaptic neuron to study its response and the conditions in which SR occurs. Our results show that new phenomena can emerge as a consequence of the interplay between the adaptive threshold and short-term synaptic processes. Concretely, this interplay induces the appearance of a second resonance peak at relatively high frequencies, which coexists with the standard SR peak located at low frequencies. The coexistence of these two resonance peaks allows the system to efficiently detect incoming weak signals for two well defined network noise levels. The precise frequency at which each one of these two resonance peaks appear is determined by the particular values of the relevant parameters involved in the dynamics of the synapses. Our main results are confirmed by employing a more realistic FitzHugh-Nagumo (FHN) neural model (which possesses an intrinsic adaptive threshold mechanism), as well as by considering more realistic stochastic synaptic models and weak input signals constituted by poissonian spike trains.

## Materials and Methods

**Figure 1:**
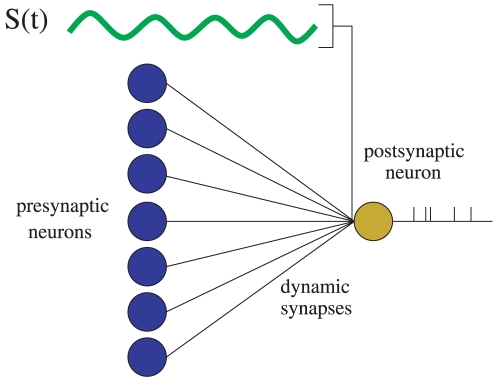
Schematic plot of the system considered in our study. The postsynaptic neuron (in yellow) receives a weak input periodic signal, and it is exposed to noisy background activity of other neurons (in blue). These neurons transmit Poissonian spike trains of frequency 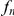 through dynamic synapses. The aim is to determine how the properties of these synapses can influence the detection of the weak signal by a postsynaptic neuron having nonlinear membrane excitability properties.

The system under study is schematized in figure 1. It consists of a postsynaptic neuron which receives both a slow, weak external signal - for simplicity, considered periodical - and the uncorrelated activity of a network of 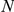 excitatory neurons. The membrane potential 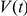 of the postsynaptic neuron is assumed as in the IF neuron model, namely
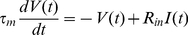
(1)where 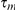 is the membrane time constant, and the neural input or excitatory postsynaptic current (EPSC) is given by 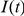, which is multiplied here by the input resistance 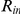. Due to the input current 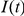, the membrane potential 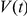 depolarizes, and when it reaches a certain threshold 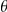, an AP is generated. The membrane potential is then reset to its resting value 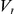 for a short period of time, called the absolute refractory period, namely 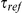.

We also assume that the neural input consists of two terms, namely 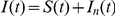. The first term, 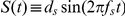, is the input weak signal, with frequency 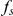 and amplitude 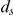. The second term is the total synaptic current generated by 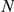 uncorrelated presynaptic neurons, namely 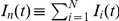. This accounts for the noisy current induced by the other neurons in the network, and its level is controlled by the mean firing rate of the network 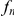. This noisy current involves an activity-dependence of the synaptic strength as proposed in a phenomenological model presented in [29]. According to this model, the state of the synapse 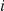 is governed by the system of equations
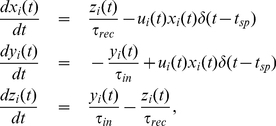
(2)where 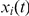, 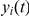, 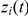 are the fraction of neurotransmitter in a recovered, active and inactive state, respectively (see [29] for details). Here, 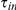 and 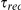 are the synapse inactivation and active neurotransmitter recovery time constants, respectively. The delta functions in equation (2) take into account that an AP arrives to the synapse at some fixed time 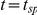. On the other hand, 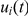 is an auxiliary variable such that 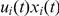 stands for the fraction of available neurotransmitter that is released after the arrival of a presynaptic AP at time 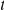 or, from a probabilistic point of view, the neurotransmitter release probability at that time. Synaptic facilitation is introduced by considering the following dynamics for 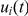:
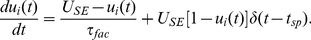
(3)This equation considers the influx of calcium ions into the neuron near the synapse through voltage-sensitive ion channels. These ions usually can bind to some acceptor which gates and facilitates the release of neurotransmitters [16]. Pure depressing synapses correspond to 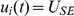 constant (which is also obtained in the limit 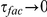), where 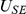 is the neurotransmitter release probability without the facilitation mechanism. Within this model, the excitatory postsynaptic current generated in the synapse 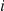 is considered to be proportional to the amount of active neurotransmitter (i.e., that which has been released into the synaptic cleft after the arrival of an AP), namely 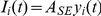.

As can be easily checked in equations (2–3), in activity dependent or dynamic synapses, the degree of synaptic depression and facilitation increases with 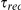 and 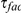, respectively, and these levels are also controlled by 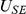. On the other hand, *static* synapses (i.e., when synapses are not activity dependent) are obtained for 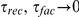.

To complete the description of the system, we assume that the firing threshold 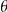 of the postsynaptic neuron follows a simple linear dynamics given by
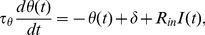
(4)where 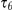 is the threshold variation time scale and 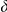 is a small positive constant. This dynamics implies that, in steady state conditions, the firing threshold displays approximately a linear dependence with the steady state postsynaptic membrane potential 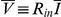, with 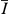 being the steady state EPSC. As explained above, this property has been observed in many neural media, and it is known as adaptive threshold dynamics [19], [20], [24], [27] (the linear dependence given by such dynamics also holds for more realistic neuron models, as we will show later on). To obtain a slow threshold dynamics as is reported in [19], we set 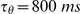 (although other values are also possible and yield the same results for our study). The parameter 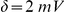 ensures that the firing threshold lies above the mean membrane potential 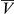, and it guarantees that the output spiking activity is driven by the current fluctuations which lead to fast depolarizations [19]. Moreover, we assume that the signal 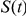 is too weak to have an appreciable effect on the value of the threshold, and therefore we set 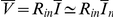 in equation (4). It is worth noting here that other threshold dynamics can be used without affecting our results, as long as the steady state linear dependence between the threshold and the input (as observed in [19]) is preserved. To ensure physiological values of the neuron threshold, we impose a minimum value for the firing threshold 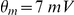. In the following, unless specified otherwise, we choose for the other parameters the values 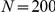, 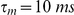, 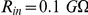, 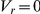, 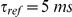, 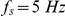, 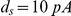, and 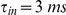, which are within the physiological range for cortical neurons.

## Results

**Figure 2:**
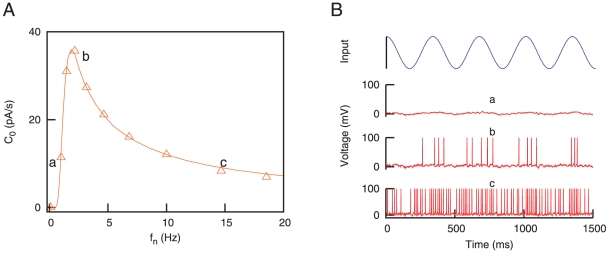
Stochastic resonance with static synapses. (A) Characteristic curve of SR as a function of the mean network rate 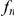. Numerical simulations of the model (symbols) agree with our mean-field theory (solid line). (B) The input signal and several time series (marked as a, b, c) of the postsynaptic membrane potential 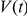 for different input noise frequencies. This is for static synapses (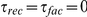), 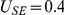, 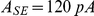, 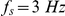 and a fixed threshold 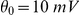.

As we have mentioned before, the phenomenon of stochastic resonance has been measured in neurons under different conditions and, in particular, in the cortex [13]–[15], [30]. Using our IF neuron model with adaptive threshold, we studied the level of background noisy activity received by a postsynaptic neuron which improves its ability to detect an incoming weak signal. This signal is considered weak in the sense that, if the level of noise is zero or sufficiently low, the neuron does not generate APs strongly correlated with the signal [31]. In order to quantify the level of coherence between the input signal 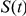 and the response of the postsynaptic neuron, we employed a cross-correlation function defined as in [32], that is,
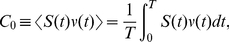
(5)where 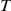 is the total recording time of each trial, typically much greater than the signal period 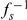, and 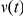 is the instantaneous firing rate of the postsynaptic neuron. This type of cross-correlation functions have been extensively used in the literature to measure the input-output dependence in neuron models and experiments (see, for instance, [11], [32], [33]). An example of stochastic resonance in the case of a presynaptic population with static synapses is shown in figure 2. For low noise frequencies, the neuron is not able to fire, and therefore, to detect the weak signal. This is reflected in the fact that 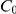 takes low values. However, when the noise frequency is increased, both noise and signal terms contribute to make the system follow the signal, that is, the neuron response becomes highly correlated with the stimulus. As a consequence of this, a maximum value of 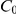 is reached. Beyond that point, the activity of the presynaptic neurons produces a high and noisy postsynaptic response, which impedes the postsynaptic neuron to detect the weak signal, and therefore the cross-correlation function 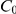 decays with its characteristic shape.

**Figure 3:**
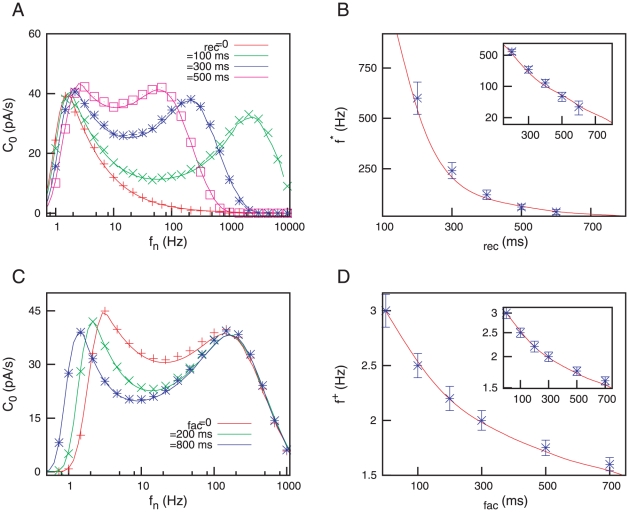
Bimodal resonances for the case of dynamic synapses. (A) Bimodal SR curves for several values of 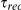, considering 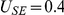 and 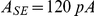. This shows that the effect of STD in stochastic resonance is the production of a second resonance peak at certain frequency 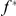 which decreases when 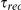 is increased. This is illustrated in panel (B), where the inset is a semi-logarithmic plot of the same data. (C) Bimodal SR curves for different values of 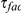, with 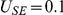 and 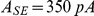. The panel (D) illustrates a decrease of the frequency 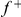 at which the first resonance peak appears as 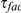 is increased. The inset in (D) is a semi-logarithmic plot of the same data. In all panels, data from numerical simulations (denoted with symbols) show a good agreement with mean-field predictions (denoted with lines).

This typical resonance behavior appears when synapses do not show any fast variability in their strength, or when the variation is only due to a slow learning processes, which we do not consider here. However, we must take into account that actual synapses show activity-dependent variability at short time scales, and this feature could modify the response of the postsynaptic neuron to the signal. In particular, since STD is a mechanism that usually modulates the high frequency inputs, one can wonder about its effect in the SR curve. In fact, our results show that this effect is quite noticeable as can be viewed in figure 3A. The figure shows the emergence of *bimodal* resonances in the presence of STD. More precisely, in addition to the standard SR peak, a second resonance peak appears at high frequencies and moves towards lower frequency values as the degree of depression increases. This second peak allows the system to efficiently detect the weak input signal among a wide range of high frequencies (note the logarithmic scale on 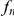). Therefore, this new resonance peak reflects that the neuron is able to properly detect the incoming signal for both low and high values of the mean network rates.

We also observe that the location of the second resonance peak has a nonlinear dependence with 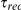. To better visualize this effect we plot in figure 3B the behavior of 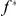, defined as the noise frequency value at which the second resonance peak is located, as a function of 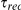. We can observe in this figure that data from numerical simulation agrees with our mean-field prediction. In the following and unless specifically specified, we have considered a time window of 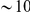 seconds for the simulations of the SR curves, and we have averaged each data point over 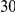 trials.

As well as STD, the facilitation mechanism is able to modulate the intensity of the postsynaptic response in a nonlinear manner for given presynaptic conditions. Following a similar reasoning to the one considered above, we expect synaptic facilitation to have and important effect in the signal detection properties of the postsynaptic neuron under noisy conditions. This effect is shown in figure 3C where, depending on the value of 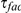, the resonance peak located at low frequencies can be tuned among different values of 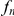. It is worthy to note that the appearance of the low frequency peak is not induced by the presence of depression or facilitation mechanisms in the synapse, since it also appears for static synapses (see figure 2A). Therefore, it corresponds to the standard SR phenomena observed in many excitable nonlinear systems. However, its precise location in the frequency range is influenced by STF. Concretely, since the effect of facilitation is to potentiate the postsynaptic response, one should expect that levels of noise which are too low to cause high 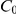 values with static synapses would, in the presence of STF, contribute to the resonance. On the other hand, the noise frequency values which were optimal to cause SR in absence of STF, becomes too high in the presence of STF and provoke a decrease in 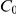. Considering these two effects together, one should expect a displacement of the first resonance peak towards lower values of 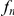 as 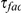 increases, which is what we observe in simulations. Since the position of the first peak is highly sensitive to the value of 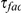, STF could have an important role for a precise discrimination of the network noisy activity level needed for the optimal detection of weak signals. The second peak, which is mainly caused by the depression mechanism, does not change its position when 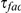 is varied, due to the prevalence of the STD effect over the STF at high frequencies. The dependence of the position of the low frequency peak, namely 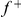, with the facilitation characteristic time is shown in the figure 3D.

**Figure 4:**
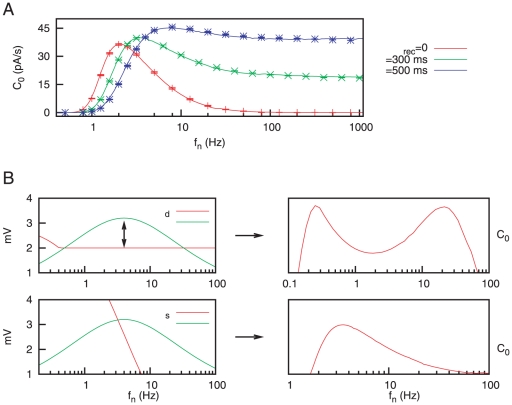
The importance of the adaptive threshold. (A) SR curves for an IF neuron model with fixed threshold 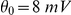 receiving a weak signal and a noisy input modulated by depressing synapses, for 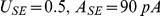 and different values of 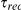. This shows that ignoring the adaptive threshold can lead to drastic modifications in the performance of the system (cf. figure 3A, see also the main text). Numerical simulations (symbols) are consistent with our simple mean field approach (lines). (B) Schematic plot to illustrate how a resonance peak appears when the amplitude of the voltage variations induced by synaptic current fluctuations (that is, 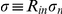) is comparable to 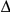 (see main text). In the case of an IF neuron model with adaptive threshold and in the presence of dynamic synapses, this occurs at two frequency values separated by a frequency range where 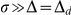 (which induces sustained spiking activity and therefore decreases the coherence 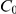 between the two maxima). For an IF neuron model with fixed threshold, however, 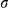 is comparable to 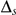 only for a single frequency value which explains the emergence of a single resonance peak.

The appearance of these bimodal resonances is not exclusively due to the dynamical characteristics of synapses. Considering adaptive thresholds is of vital importance for the emergence of bimodal resonances. To illustrate this, we have computed SR curves for different values of 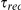 and an IF neuron with fixed firing threshold (that is, an input-independent threshold), namely 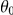. The result is shown in figure 4A, where one can see that STD is not able to induce a second resonance peak when neuron threshold is considered independent of the mean membrane potential. Instead of this, we found that 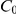 does not decay from its peak value to zero for high 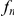 values, but it stabilizes at a steady value 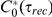. Such high steady value means that some level of coherence between the weak signal and the postsynaptic response is maintained for arbitrarily high mean rates. It is worthy to note that, for a particular value of 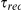 (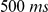 in the figure), the value of 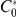 obtained is similar to its peak value, thus allowing a good detection over a wide range of background firing rate values.

This saturation of 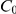 for strong enough STD, which is due to the oversimplification assumed by the IF model with fixed threshold, can be easily explained as follows. Firstly, our simulations show that, in order to have large values of 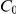, a necessary condition is that 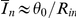, with 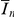 being the mean noisy input current (if 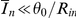 the postsynaptic neuron is not firing at all, and if 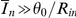 the postsynaptic neuron is firing all the time). Secondly, in the presence of STD and for high background noise rate, the mean noisy input current 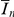 saturates at certain value 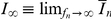 – see expressions for the mean and peak value of the postsynaptic current in Text S1 – which is infinity for 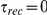 and decreases as 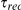 increases. Moreover, for 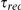 sufficiently high (strong depression), the mean noisy current is near to its asymptotic value 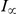, for a finite and relatively low noise frequency 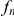. As a consequence, there is a sufficiently high value of 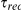 for which 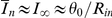. In this situation an optimal 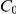 value will be maintained over a wide range of network firing rates, as the figure 4A shows.

Since short-term synaptic mechanisms alone are not able to induce bimodal resonances in simple IF neurons with fixed threshold, as we have already seen, a plausible hypothesis is that this two-peak resonant behavior emerges from the interplay between these synaptic mechanisms and adaptive thresholds. To illustrate this hypothesis, we can sketch a simple explanation of such cooperative effect by considering that, for an excitable system displaying SR, a resonance peak is obtained when the strength of the fluctuations is approximately equal to some potential barrier height [34]. According to previous works [32], [34], and considering the potential function associated with the IF model (see, for instance, [35]), one can easily demonstrate that the condition for the appearance of a resonance peak is 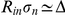, where 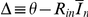 is the distance in voltage between the mean membrane potential and the firing threshold. Considering a threshold dependence such as the one defined in equation (4) in the steady state, 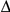 takes the value of a small constant (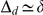, where the subindex 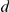 highlights that it corresponds to a dynamic threshold situation) for large enough 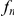. Since the dependence of 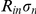 with 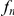 is non-monotonic for dynamic synapses (see Text S1 for details), plotting together the expressions of 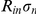 and 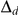 as a function of 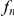 shows two well located crossing points, as the top panels of figure 4B illustrate. Each one of these crossing points is associated then with a maximum in 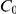 (as we have argued above), and therefore a bimodal resonance is obtained. The local minimum in 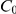 is due to a high number of erratic firings of the postsynaptic neuron, which is caused by high values of the current fluctuations (compared with 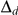) around the point where the local minimum appears. This feature is depicted in the top-left panel of figure 4B with a double-head arrow. Without such large current fluctuations, the local minimum of 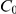 would vanish and the bimodal resonance would be lost. On the other hand, for the case of an IF neuron with fixed threshold, the quantity 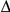 (namely 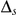, to highlight that it corresponds to a fixed threshold situation) is a monotonically decreasing function of 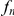. In these conditions, a single crossing point between 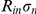 and 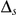 is obtained, and therefore the SR curve presents a single peak, as the bottom panels of figure 4B show. It should be noted that, for certain sets of values of the model parameters, two crossing points between the level of EPSC fluctuations and 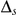 can also be found for a fixed neuron threshold. However, in such situations 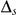 is large and comparable to 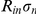. As a consequence, the local minimum of 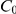 cannot be obtained, and the SR curve remains with the characteristic single-peak shape.

**Figure 5:**
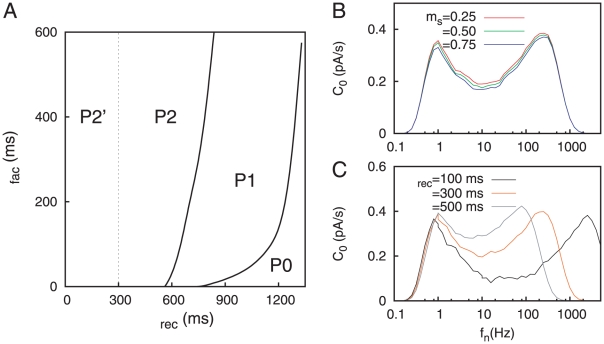
Phase diagram and more realistic signals. (A) Phase diagram, obtained from our mean-field approach, which shows different regimes of the behavior of the system, for 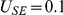 and 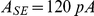. Labels P0, P1, P2 denote, respectively, regions in which zero, one, or two resonance peaks occur. The region P2′ denotes values of the synaptic parameters for which a second resonance appears, but at a frequency too high to be considered realistic (that is, 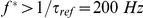). For 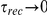 the typical single resonance peak is recovered. (B) Bimodal resonances obtained for a realistic signal (a rate-modulated poissonian spike train influenced by STD mechanisms), with 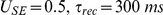, 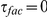 and different values of the modulation amplitude. (C) Same as panel B, but for 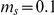 and different values of 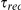.

The appearance of bimodal resonances gives a high versatility to neurons as weak signals detectors. In actual neural media, populations of neurons could take advantage of such versatility, and they could use the high heterogeneity of synaptic properties [36] to organize groups of neurons with non-resonance, single-resonance or two-resonance peak behavior. A phase diagram, which locates the repertoire of different behaviors in the space of synaptic relevant parameters, is shown in figure 5A. For realistic synaptic conditions, the three types of behavior are accessible. The region P2′ corresponds initially to two resonances, but the second resonance is usually located in an extremely high network rate (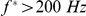), which means that the second resonance does not occur in realistic conditions. If we increase 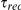 (for a given value of 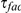), the system pass from a single-peak resonance behavior (region P2′) for low 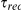, to the bimodal resonance phase P2 (because increasing 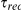 implies lowering 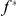). After that, the system reaches a single-peak behavior again (due to the fusion of the two peaks of the bimodal resonance into just one peak, namely region P1). Finally, increasing 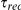 even more would lead to a decrement of the detection ability of the neuron, leading to the zero-resonance phase (labeled as P0 in the figure).

### Robustness of the results

The fact that we considered a simplified neuron model allowed us to make an analytical treatment, which confirmed the numerical results both for STD and STF, as we have already seen. However, we should consider whether bimodal resonances appear in more realistic conditions. For instance, so far we assumed (as a first approximation) that the signal term was a sinusoidal function of weak amplitude and slow frequency. It is well known, however, that *in vivo* neural signals are usually encoded in spike trains. Moreover, since these presynaptic spike trains affect the postsynaptic neuron via the synapses, STD mechanisms should, *a priori*, affect the signal term as well. To take into account this possibility we can consider, for instance, a signal constituted by a poissonian spike train whose instantaneous firing rate is modulated by a slow sinusoidal function [37]. Therefore, in order to test our results in more realistic conditions, we consider a signal term given by 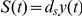, where 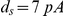 is the amplitude of the signal, and 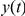 introduces STD on the signal (see equation (2)). As it has been previously mentioned, we also assume a poissonian train of spikes with instantaneous firing rate 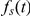 given by
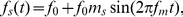
(6)where 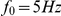 is the time-averaged firing rate, and 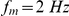 and 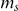 are the frequency and amplitude of the modulation, respectively. The consideration of this more realistic signal term does not have a dramatic effect on the resonant behavior of the neuron, as can be seen in figures 5B and C. Indeed, STD induces the appearance of a second resonance peak for different values of the amplitude modulation 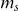 (figure 5B), and it also appears for the same range of values of 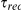 and 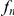 than before (figure 5C), which implies that our results are robust with this more realistic assumption for the signal term.

**Figure 6:**
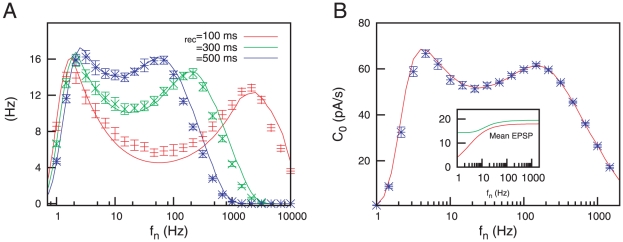
Additional input-output measurements and more realistic adaptive threshold. (A) SR curves for an IF neuron model with adaptive threshold receiving a weak sinusoidal signal and uncorrelated noisy input modulated by depressing synapses. The input-output measurement employed is the amplitude of the modulation of the postsynaptic firing rate, namely 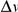. Numerical simulations (symbols) confirm the mean field prediction (lines), and highlights the robustness of our results when one considers other input-output measurements. Parameters are 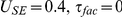 and 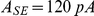, and results have been obtained after averaging over 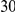 different systems. (B) Bimodal resonances for an IF neuron whose adaptive threshold compensates the increments of 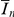 only partially (see main text for details). The relation 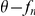, needed to obtain the SR curve, has been slightly smoothed to allow a better visualization of the first resonant peak (see the inset). Parameter values are 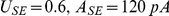, 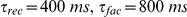, 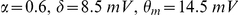 and 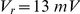.

Another important question to check the robustness of our results is whether bimodal resonances also appears when one considers other input-output measurements. A typical measurement which may be employed here is the amplitude of the modulation of the postsynaptic firing rate caused by the sinusoidal signal. For this purpose we define the amplitude of the firing rate modulation as 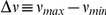, where 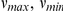 denotes, respectively, the maximum and minimum value of the postsynaptic firing rate measured by averaging over a high enough number of systems. As expected, 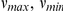 correspond to the value of 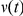 at times when 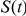 is maximal and minimal, respectively. The figure 6 shows that bimodal resonances are obtained in the same conditions as before, that is, when the synapses present short-term plasticity mechanisms (here, short-term depression) and the postsynaptic neuron displays adaptive threshold mechanisms. The emergence of bimodal resonances is, therefore, quite robust and can also be obtained by using other input-output measurements, such as the signal-to-noise ratio defined in [34] (data not shown).

We should clarify that, so far, we have considered that the firing threshold 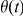 is able to fully compensate any increment in the mean excitatory postsynaptic current 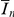. This can be achieved by setting 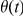 into its steady state value (that is, assuming 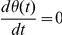 in equation (4)), which yields 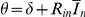, meaning that any increment in 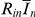 will cause an increment of the same magnitude in 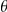. However, this could not always be the case, as experimental data reported in [19] indicate that an increment in the mean input 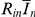 causes a smaller increment in 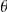. Then, to account for this finding and in order to test the main predictions of our study, we have introduced a constant factor 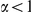 in the last term of the right hand side of equation (4), so that in steady state conditions one has 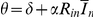 as suggested by experiments in [19]. The figure 6B shows that bimodal resonances also appear when one takes into account this realistic consideration about adaptive threshold, highlighting the robustness of our results. In this figure, the parameter values of the adaptive threshold are set to 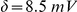 and 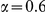, which are close to the physiological values measured in [19]. Both analytical and numerical results also indicate that a higher value of the reset membrane potential 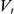 is convenient to preserve the bimodal resonances for these conditions. Despite all these results, further research employing more realistic models of adaptive threshold are needed to gain more knowledge about this important topic.

**Figure 7:**
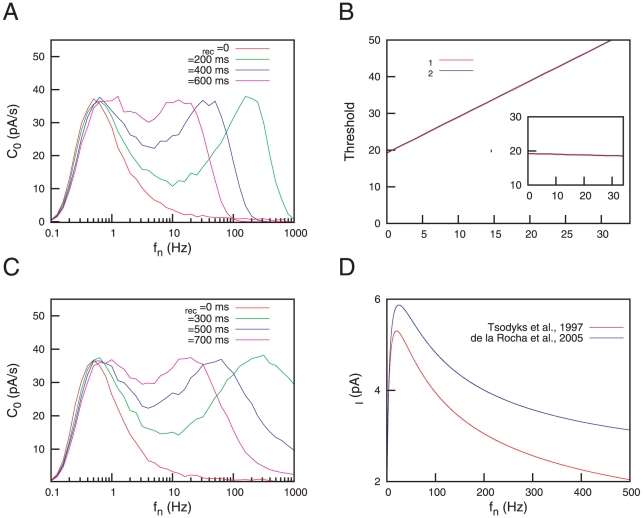
More realistic neuron and synapse models. (A) Numerical SR curves for a postsynaptic FHN neuron model receiving a weak signal and uncorrelated background noisy activity of frequency 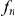, for 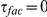, 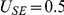, 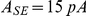 and different values of 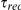. In order to estimate the firing times of the FHN model, the dynamics of the variable 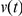 was thresholded at 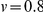. (B) Estimation of the neuron firing threshold for different values of the constant input current 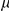, and employing two different measures (see the main text for details). The inset shows that the quantity 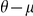 slowly decreases with 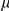 (as occurs in actual neurons), although it may be considered approximately constant for the range of values of 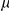 considered. (C) Numerical SR curves for several 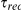 values and 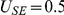, when a more realistic stochastic model for the synapses is employed (see main text). We set the parameters of the stochastic model in 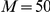, 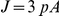, 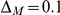 and 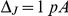. (D) Comparison of the standard deviation of the synaptic current for the two synaptic models employed in our study. The conditions are the same than those in panel C but 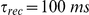. Although the difference between the predictions of these two models is about 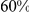 for high frequencies, similar bimodal resonances are obtained in both cases.

The emergence of bimodal resonances is also maintained when one considers a more realistic neuron model to simulate the response of the postsynaptic neuron. Although we have employed an adaptive threshold to include some of the nonlinear features of actual neurons into the IF neuron model, it should be convenient to test our findings by considering an intrinsic nonlinear neuron model which could present this type of threshold variability without additional ingredients. A common simple model employed in the literature to describe the nonlinear excitability properties of actual neurons is the FitzHugh-Nagumo neuron model [38], which can be defined as
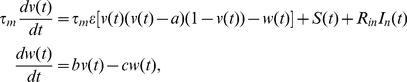
(7)where 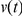 represents the postsynaptic membrane potential, 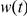 is a slow recovery variable related with the refractory time, and 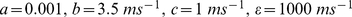 are parameters of the model. With this choice of values for the parameters, the model is set in the excitable regime, the (dimensionless) voltage 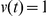 corresponds to 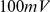 and time is given in 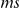. We also consider 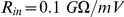 and 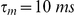, which lie within the physiological range of actual cortical neurons. The terms 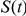 and 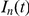 are described as before, with 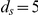. We have performed numerical simulations of the system presented in figure 1, but considering now this FHN model for the membrane potential of the postsynaptic neuron. The results are shown in figure 7A, which illustrates that for large enough values of 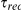 a bimodal resonance also appears. The location of the second peak moves towards lower values of 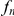 as 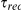 increases, as it was found with the IF model with adaptive threshold. The range of values of the noisy frequency 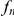 at which the second peak is located is also the same than in the previous model.

It is necessary to demonstrate here that the FHN model presents several threshold variability properties which are similar to those we assumed for the IF neuron model with adaptive threshold. In order to check this, we define two types of temporal stimuli that the postsynaptic neuron can receive (in addition to the weak signal): 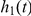 and 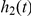. The first stimulus, 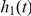, consists in a train of narrow (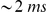) square pulses of frequency 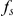 (that is, the same frequency as the signal). We impose that each one of these pulses arrives to the postsynaptic neuron every time the signal 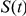 reaches its maximum value, namely 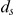. Similarly, the other type of stimulus, 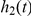, consists in a train of narrow (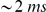) square pulses also of frequency 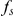, but in this case each pulse arrives at the postsynaptic neuron when 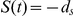 (that is, every time the signal takes its lowest value). We also set a constant input 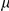, in such a way that the total input to the postsynaptic neuron is given by 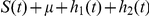. For a given fixed value of 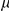, we can determine the value of the neural firing threshold by increasing the strength of the stimulus 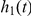 (that is, the height of the narrow pulses) until an AP is generated as a consequence of such stimulus (we denote this minimal strength of the stimulus 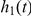 which generates APs as 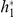). This measure of the firing threshold will be denoted as 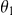. Similarly, we can perform a second estimation of the neuron threshold, namely 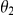, by varying the strength of 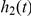 until an AP is generated in response to this second stimulus (being this minimal stimulus for the generation of APs called 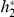). According to these definitions, the different measures of the firing threshold that we adopt here will be 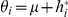, for 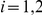. It is worth noting that, for coherence purposes with the case of the IF neuron presented above, the firing threshold is defined here as the distance between the stable fixed point of the dynamics of the FHN for zero input (that is, 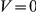) and the unstable branch of the nullcline (that is, the value of 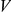 for which a spike is generated). Both estimations (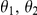) of the firing threshold, as a function of the constant input 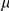, are shown in figure 7B. The figure illustrates two major features of the excitability properties of the FHN neuron model. The first one is that, independently of the value of 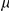, both estimations give almost identical results for the value of the neural firing threshold of the FHN neuron model. Since the only distinction between the stimuli 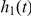 and 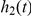 is a difference in amplitude of 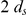, which is due to the signal term, this result indicates that the weak signal does not influence the value of the firing threshold (independently of the value of the constant input 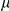). This confirms the assumption we made for the IF model in equation (4). The second major feature illustrated by the figure 7B (see also the inset of the same figure) is that the value of the firing threshold increases linearly with 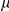. This dependence coincides with the expression for the steady state of the firing threshold obtained from equation (4), which we assumed for the IF model with adaptive threshold. Therefore, the hypothesis we made on the modeling of the threshold variability for the IF model is appropriate as is confirmed by using more realistic neuron models, such as the FHN model (which incorporates nonlinear excitability properties).

The robustness and generality of our previous results can be also tested by considering a more realistic model for the activity-dependent synaptic mechanisms. For instance, until now we have treated the synapses employing a deterministic model of dynamic synapses for the sake of simplicity. However, it is known that actual synapses have a stochastic nature [39] and their fluctuations can play an important role in neural computation [39], [40], and therefore they should be taken into account. In particular, since the SR curves depend strongly on the noise properties, it is important to consider the additional source of noise due to synaptic fluctuations, since this could lead to a very different emergent behavior in the system. To see the influence of such fluctuations in our analysis, we have simulated our system using an intrinsically stochastic model of dynamic synapses presented in [41]. This model considers that each connection between neurons has a finite number of functional contacts, or synaptic buttons, and this number is randomly chosen (for each particular connection) from a Gaussian distribution of mean 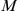 and standard deviation 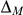. In addition, the strength of each individual synaptic button is also randomly determined from a Gaussian distribution of mean 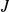 and standard deviation 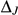. The release of a neurotransmitter vesicle from a synaptic button to the synaptic cleft, when an AP arrives at the button, is modeled as a random event. After that release, the recovering of the synaptic button is considered as a probabilistic event following a Poisson distribution with a typical time 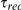. This probabilistic model gives the same mean values for the EPSC, but the fluctuations differ from the deterministic model of dynamic synapses (see figure 7D and Text S1 for more details). As it is shown in figure 7C, this stochastic model induces the same phenomenology during SR experiments as those for the deterministic model described by (2–3). That is, for the case of static synapses, a single resonance peak at low frequencies is obtained as usual, and when 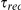 is increased, a second peak appears at high frequencies, with the resonance peak location moving towards low noise rates. We also tested our results by considering a conductance based description of the synaptic current (data not shown), leading to the appearance of bimodal resonances as well.

## Discussion

It is widely known that noise can have relevant and positive effects in many nonlinear systems in nature. These effects include noise-induced phase transitions [42], [43], stochastic dynamics of domain growth [44], or multiple types of stochastic resonance [6], [32], [34], to name a few. The particular case of stochastic resonance has been widely studied in the context of biological systems [6], [7], and in particular in the brain. More precisely, stochastic resonance phenomena could occur in many brain areas, such as the cortex [13]–[15], the hippocampus [10], or the brain stem [11]. Therefore, it is highly relevant to understand the influence that some features of actual neural systems could have in the emergence of stochastic resonance phenomena.

Short-term synaptic mechanisms are, in this framework, a good candidate to consider. It is known, for instance, that both STD and STF play an important role in the transmission of relevant correlations between neurons in noisy environments [18], in the temporal maintenance of information in persistent states of working memory tasks [45], in the recall of stored memories on attractor neural networks [46], or in the switching behavior between neural activity patterns [47], [48]. However, the interplay between these two mechanisms, or between them and other adaptation processes of neurons, has not been fully addressed yet.

In this work we have considered the role of dynamic synapses in the detection of weak signals by neurons embedded in neural networks, via a stochastic resonance formalism. To the best of our knowledge, this is the first study that shows the dramatic effect of the interplay between the dynamical nature of synapses and adaptive threshold mechanisms on the stochastic resonance properties of neurons. More precisely, we have demonstrated that this interplay may originate the appearance of bimodal resonances, where the location of the resonances in the frequency domain is related with the relevant synaptic parameters. In addition, to test our findings we have used several neuron and synapse models, as well as a number of realistic considerations such as poissonian input spike trains (for both signal and noise terms) and the consideration of an additional noisy term in the adaptive threshold dynamics (data not shown), for instance.

Although recent studies [11], [49] have also suggested a relevant role of STD in neural stochastic resonance, the emergence of bimodal resonances, which is the crucial point of our study, is missed in these works. On the other hand, bimodal resonances have been found in several complex excitable and bistable systems [50]–[58], although their occurrence in neural media has not been reported up to date, nor experimentally neither by employing realistic neural modeling.

An interesting issue to consider in future studies is, for instance, whether the interplay between short-term plasticity mechanisms and other threshold dynamics (apart from the one considered here) could also cause the appearance of bimodal resonances. In this work, we have explicitly considered a linear model for the adaptive threshold dynamics, which constitutes a good approach in steady state conditions as the experiments suggest [19]. However, more detailed models are needed if one is interested, for instance, in the effect of the interplay between dynamic synapses and adaptive thresholds in non-steady state conditions, such as in the transmission of fast transient signals. Other neural homeostatic mechanisms may be considered as well. It is known, for instance, that the postsynaptic response of a neuron may influence its own excitability properties— a property which is known as *neural adaptation*. This mechanism has been studied in a wide range of neural systems (see, for instance, [22], [23], [59], [60]) and its effect in neural dynamics could be similar (but only under certain conditions) to the adaptive threshold dynamics considered here. Therefore, analyzing in detail the interplay between neural adaptation and dynamic synapses during the transmission of weak signals constitutes an open problem and deserves future investigation.

Although we have considered in our study that the effect of the spikes arriving through 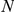 afferent synapses is a noisy synaptic current that we assume to be approximately Gaussian, more realistic conditions must consider other types of noise, including for instance the possibility of non-Gaussian noises. This has been reported to have a strong effect in stochastic resonance phenomena by enlarging the range of noise intensity at which detection occurs, and making the detection less dependent on the noise [61]. Interestingly, this effect can be also obtained in our case by modifying the level of depression (note that in the present study we use a logarithmic scale for the frequency domain, which indicates that the second resonance peak is in fact a very broad peak).

It should be also interesting to investigate whether bimodal resonances may be obtained as a combination of individual synapse-neuron properties and network mechanisms. For instance, one could assume that the role of the adaptive threshold (which compensates for the increment in the mean noisy input with the presynaptic firing rate) could also be achieved by a certain balance between the input of excitatory and inhibitory populations. In these conditions, bimodal resonances could also appear in balanced networks of simple IF neurons connected by dynamic synapses, and some preliminary findings suggest that this could be the case (data not shown). The concrete details of this possible emergence of bimodal resonances as a consequence of synaptic and network mechanisms is, therefore beyond the scope of this work, and a complete study of this interesting issue will be published elsewere.

Several questions raised by our study should be experimentally tested. An interesting prediction to test is, for instance, whether STF has the effect on the first resonance peak predicted by our results. This gives an idea of the relevance of the dynamics of intracellular calcium in processing weak signals at spontaneous activity states, which are common in cortical areas. The observed dependences of the position of the peaks with the synaptic characteristic time scales should be confirmed experimentally as well. Finally, the question of how these bimodal resonances could be measured in actual cortical structures, and its possible effect in brain cognitive tasks, constitutes an interesting issue that still remains open. In fact, the experimental validation of our work would require to select brain areas in which neurons receive at least two clearly distinguishable types of inputs (so one may identify a signal and a noise term). Good candidates to consider are sensory associative regions, in which neurons may receive distinguishable inputs from different senses [11]. Indeed, very recent works indicate that the interplay between different sensory inputs could lead to an enhanced detection of weak sensory stimuli (a phenomenon known as crossmodal resonance [11], [62]), and therefore this would constitute a proper framework to test our theoretical predictions.

## Supporting Information

Text S1 **Theoretical derivations.** In this supplementary text we derive an analytical approximation of the input-ouput correlation function defined in the main text, which is used together with numerical simulations to show the behavior of the system under study.
(PDF)
