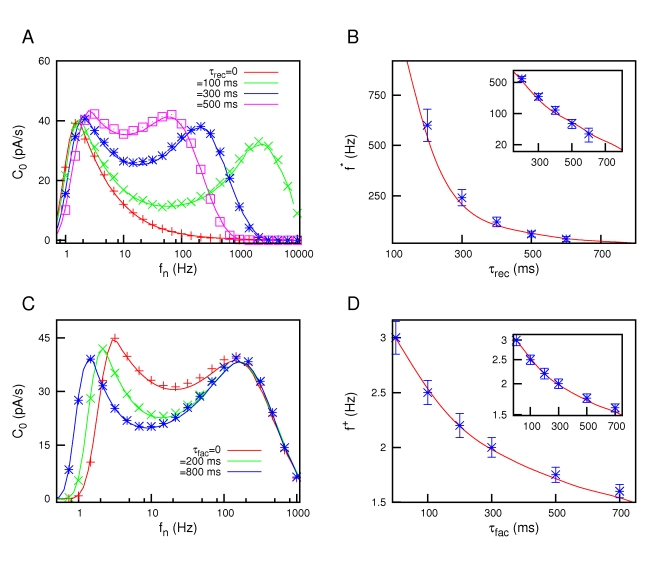# Correction: Emergence of Resonances in Neural Systems: The Interplay between Adaptive Threshold and Short-Term Synaptic Plasticity

**DOI:** 10.1371/annotation/7f822457-9765-44d2-927a-ff530bd5a2d0

**Published:** 2011-04-06

**Authors:** Jorge F. Mejias, Joaquin J. Torres

Due to a technical error, there are missing symbols in Figure 3. Please view the correct Figure 3 file here: 

**Figure:**