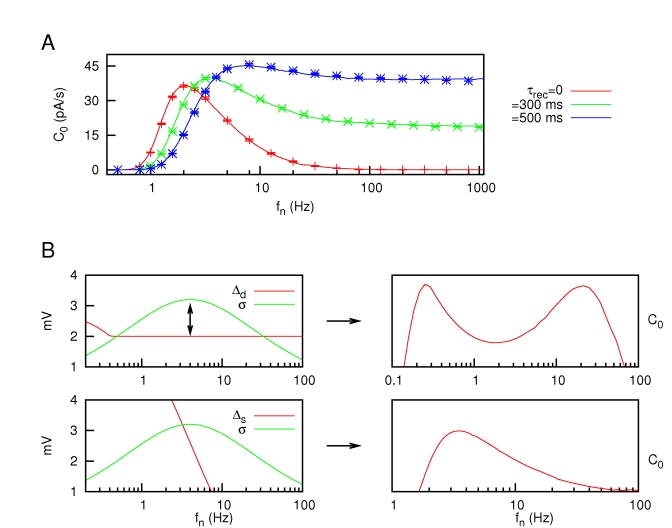# Correction: Emergence of Resonances in Neural Systems: The Interplay between Adaptive Threshold and Short-Term Synaptic Plasticity

**DOI:** 10.1371/annotation/3c57af7b-02a6-4267-b586-8b5a437fa5ba

**Published:** 2011-04-06

**Authors:** Jorge F. Mejias, Joaquin J. Torres

Due to a technical error, there are missing symbols in Figure 4. Please view the correct Figure 4 file here: 

**Figure:**