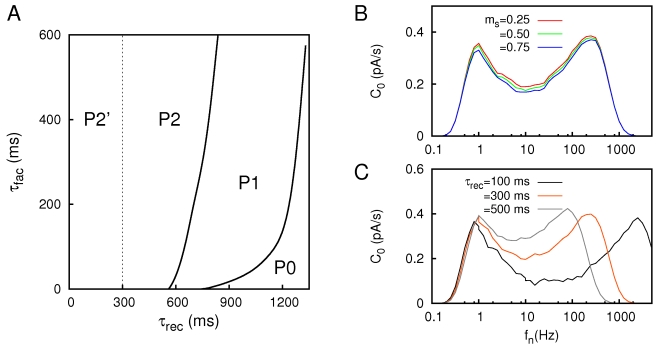# Correction: Emergence of Resonances in Neural Systems: The Interplay between Adaptive Threshold and Short-Term Synaptic Plasticity

**DOI:** 10.1371/annotation/69c2f5a6-0fcd-46c4-baaf-0766e928bfd6

**Published:** 2011-04-06

**Authors:** Jorge F. Mejias, Joaquin J. Torres

Due to a technical error, there are missing symbols in Figure 5. Please view the correct Figure 5 file here: 

**Figure:**