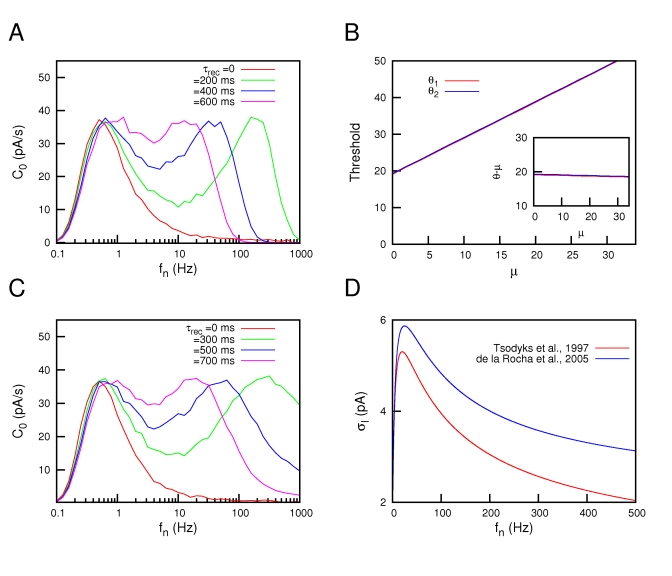# Correction: Emergence of Resonances in Neural Systems: The Interplay between Adaptive Threshold and Short-Term Synaptic Plasticity

**DOI:** 10.1371/annotation/4e6e67ff-365f-4f26-a6f9-cc9f551a089b

**Published:** 2011-04-06

**Authors:** Jorge F. Mejias, Joaquin J. Torres

Due to a technical error, there are missing symbols in Figure 7. Please view the correct Figure 7 file here: 

**Figure:**