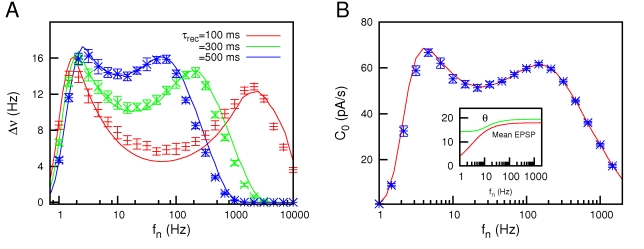# Correction: Emergence of Resonances in Neural Systems: The Interplay between Adaptive Threshold and Short-Term Synaptic Plasticity

**DOI:** 10.1371/annotation/1fe001e2-eb2a-4891-a947-a0105812911b

**Published:** 2011-04-06

**Authors:** Jorge F. Mejias, Joaquin J. Torres

Due to a technical error, there are missing symbols in Figure 6. Please view the correct Figure 6 file here: 

**Figure:**