# Supplementary material for: Emergence of Resonances in Neural Systems: The Interplay between Adaptive Threshold and Short-Term Synaptic Plasticity
Source: PLoS One. 2011 Mar 8;6(3):e17255. doi: 10.1371/journal.pone.0017255 (PMC3050837; doi:10.1371/journal.pone.0017255)
Supplement: Text S1 — Theoretical derivations. In this supplementary text we derive an analytical approximation of the input-ouput correlation function defined in the main text, which is used together with numerical simulations to show the behavior of the system under study. (PDF) [file pone.0017255.s001.pdf]

# Supplementary material

Here we derive the analytical expression for the cross-correlation measure $C_0$ between the response of the postsynaptic neuron and the weak input signal, in the presence of noisy activity. First, we obtain the expressions for the noisy EPSC with dynamic synapses, for both the deterministic model and the stochastic model. After that, we will obtain the expression for the mean firing rate of the IF postsynaptic neuron in the presence of such noisy EPSC, and we will use this expression to obtain a mean-field formula for $C_0$.

We consider a population of $N$ presynaptic neurons firing uncorrelated Poisson spike trains at a certain frequency $f_n$. We also assume that the synaptic current $I_i(t)$ generated by an AP arriving at time $t^*$ in a particular synapse $i$ is proportional to the fraction of active neurotransmitters in that synapse, namely, $y_i(t)$ – cf equation 2 of the main text. In this situation the postsynaptic current at time $t = t^* + \tau$ is given by

$$I_i(\tau, t^*) = I_p \exp(-\tau/\tau_{in}). \quad (1)$$

where $I_p$ is the peak value of the EPSC, reached at time $t = t^*$. Considering a stimulation with a stationary Poissonian AP train, the peak value $I_p$ can be substituted by an averaged stationary EPSC amplitude. One easily obtains from equations (2-3) of the main text that

$$I_p = A_{SE}\ u_\infty x_\infty \quad (2)$$

where $u_\infty$ and $x_\infty$ are, respectively, the facilitation and depression variables in the stationary state, and their expressions are given by

$$u_\infty = \frac{U_{SE} + U_{SE}\ \tau_{fac}\ f_n}{1 + U_{SE}\ \tau_{fac}\ f_n}, \quad (3)$$

$$x_\infty = \frac{1}{1 + u_\infty\ \tau_{rec}\ f_n}. \quad (4)$$

Using the fact that $N$ is large enough, the mean current of the presynaptic population and its fluctuations are given by

$$\overline{I}_n = N f_n \tau_{in} I_p \quad (5)$$

$$\sigma_n^2 = \frac{1}{2}\ N f_n \tau_{in} (I_p)^2 \quad (6)$$

where we assumed that $\tau_{in} \ll \tau_{rec}$. Equations (5) and (6) allow to characterize the noisy input from the presynaptic neurons. The dependence of these quantities with $f_n$ is shown in the figure 1 of this supplementary material. It is worthy to note that, although we have assumed a poissonian distribution for the spike trains, the mean-field approach considered here holds for other distributions of the spike trains [1], as long as presynaptic neurons remain uncorrelated in time and their number is large enough.

We can also consider the more realistic model of synaptic transmission presented in [2], which takes into account the stochastic nature of synaptic release events. Following [3], this model gives the same value for the mean current but yields an expression for the EPSC fluctuations (for an uncorrelated noisy input) that is given by

$$\sigma_n^2 = NMJ^2 u_\infty x_\infty f_n \left[ 1 + \Delta_J^2 + \frac{u_\infty \left[ M(1 + \Delta_M^2) - 1 \right]}{1 + u_\infty \tau_{rec} f_n (1 - u_\infty/2)} \right]. \quad (7)$$

Here, $M$ is the number of synaptic functional contacts, $J$ is the synaptic strength per functional contact, and $\Delta_J$, $\Delta_M$ are their respective standard deviations.

With these expressions (taking the fluctuations either from the deterministic or from the stochastic model), one can obtain the mean firing rate of the postsynaptic neuron by solving the Fokker-Planck equation associated with the dynamics of the membrane potential [4, 5]. We define the quantities

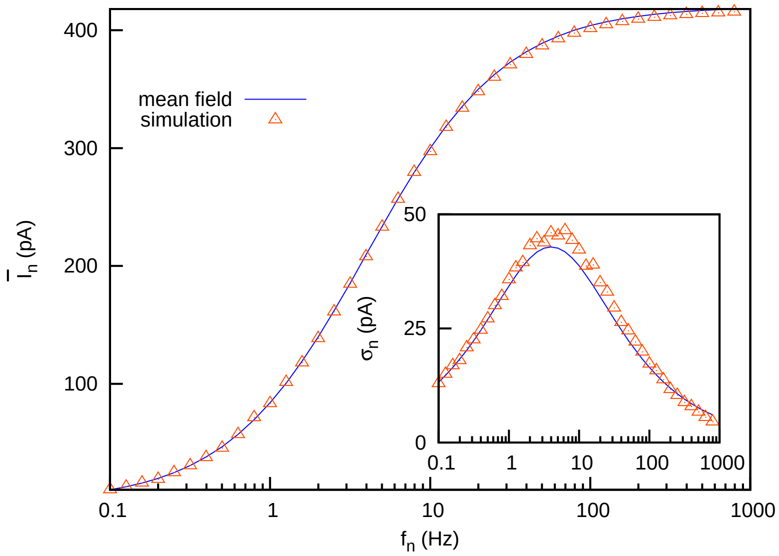


**Figure 1. Excitatory postsynaptic current with dynamic synapses.** Mean EPSC as a function of the mean firing rate $f_n$, with $U_{SE} = 0.5$, $A_{SE} = 70\ pA$ and $\tau_{rec} = 500\ ms$. Numerical simulations (symbols) are supported by mean field results (solid lines). In the inset, we can see the good agreement between mean field and simulations for the EPSC fluctuations.

$$y_\theta(t) = \frac{\theta - R_{in}\overline{I}_n + S(t)}{R_{in}\sigma_n} \tag{8}$$

$$y_r(t) = \frac{V_r - R_{in}\overline{I}_n + S(t)}{R_{in}\sigma_n}, \tag{9}$$

and assume that the weak signal $S(t)$ evolves slowly compared with the neuron dynamics. The firing rate of the postsynaptic neuron is then given by

$$\nu(t) = \left[\tau_{ref} + \tau_m \int_{y_r(t)}^{y_\theta(t)} dz\sqrt{\pi}\exp(z^2)(1+\mathrm{erf}(z))\right]^{-1}. \tag{10}$$

For the case in which we have an adaptive threshold, we set $\frac{d\theta(t)}{dt} = 0$ in equation (4) of the main text to obtain the steady state value $\theta = \delta + R_{in}\overline{I}_n$, with $\overline{I}_n$ given by equation (5). On the other hand, for the fixed threshold approach we simply set $\theta = \theta_0$. Equation (10), together with the expressions of the EPSC and the threshold conditions obtained above, allows to evaluate equation (5) of the main text and to obtain

$$C_0(\nu) = \int_0^{1/f_s} f_s d_s \sin(2\pi f_s) \left[\tau_{ref} + \tau_m \int_{y_r(t)}^{y_\theta(t)} dk\sqrt{\pi}\exp(k^2)(1+\mathrm{erf}(k))\right]^{-1} dt, \tag{11}$$

where we have set $T = 1/f_s$. By evaluating numerically this expression, one obtains analytical curves for the input-output correlation which can be compared with the results from numerical simulations of the system described in the main text.

## References

1. Mejias JF, Torres JJ (2008) The role of synaptic facilitation in spike coincidence detection. J Comput Neurosci 24: 222-234.

2. de la Rocha J, Parga N (2005) Short-term synaptic depression causes a non-monotonic response to correlated stimuli. J Neurosci 25: 8416-8431.

3. de la Rocha J, Moreno R, Parga N (2004) Correlations modulate the non-monotonic response of a neuron with short-term plasticity. Neurocomp 58-60: 313-319.

4. Tuckwell HC (1989) Introduction to theoretical neurobiology. Volume 2: nonlinear and stochastic theories. Cambridge.

5. Brunel N (2000) Dynamics of sparsely connected networks of excitatory and inhibitory spiking neurons. J Comp Neurosci 8: 183–208.
